# Rituximab for Steroid-Dependent Minimal Change Disease in Adults: Is It Time for a Change?

**DOI:** 10.7759/cureus.22313

**Published:** 2022-02-17

**Authors:** Lakshmi Kannan

**Affiliations:** 1 Nephrology, Pikeville Medical Center, Pikeville, USA

**Keywords:** complications’, steroid dependent, rituximab, kidney biopsy, minimal change disease

## Abstract

Minimal change disease (MCD) is a common cause of nephrotic syndrome, and steroid treatment is usually effective at the expense of adverse effects and frequent relapses. Rituximab, a monoclonal antibody against cluster of differentiation (CD)20 B-lymphocytes, leads to depletion of B-cells and has been frequently used to treat relapsing MCD in children. The efficacy of rituximab in treating adult MCD is limited. We report our experience with the use of rituximab for adult biopsy-proven MCD.

Our series includes four adult patients (two males and two females), aged 22-80 years, treated with rituximab. All four patients achieved a complete remission with rituximab which lasted from 12 to 19 months. No adverse events from rituximab were observed. This shows the remarkable efficacy of rituximab in the treatment of minimal change disease in adults and may be preferred in patients at high risk for the development of adverse events from corticosteroids.

## Introduction

Minimal change disease (MCD) is the most common primary nephrotic syndrome in children but also accounts for 15% of adult nephrotic syndrome, of which 25% have a frequently relapsing course and 30% become steroid dependent [1]. Glucocorticoids are recommended as the first-line therapy for MCD by the Kidney Disease: Improving Global Outcomes (KDIGO) [2], as the response rate is reported to be 75% [3].

Long-term steroid therapy comes with adverse clinical effects such as steroid-induced hyperglycemia, increased salt and water retention leading to hypertension and increased cardiovascular events, dyslipidemia, decreased bone mineralization, and psychiatric disease [4]. And, MCD with frequently relapsing cases (about 56-76% of cases) is medically challenging [5].

Several alternate therapeutic approaches have been attempted, but an alternate treatment for establishing rapid induction, long-term remission with few adverse events is still being established. Immunosuppressive agents such as calcineurin inhibitors (CNI), cyclophosphamide, and mycophenolate mofetil have been used but are also limited by frequent relapses or adverse events such as acute kidney injury with CNI, gastrointestinal side effects from mycophenolate, and infertility with cyclophosphamide [6].

Rituximab is a chimeric murine/human monoclonal immunoglobulin antibody that targets cluster of differentiation (CD)20, a B-cell differentiation marker [7]. It has been used in children since 2006 to treat frequently relapsing MCD [8]. However, the remission rate after rituximab in adults remains undetermined. Here, we describe the effects of rituximab on the induction of adult patients with MCD who frequently relapsed with glucocorticoids.

## Case presentation

Case 1

**Figure 1:**
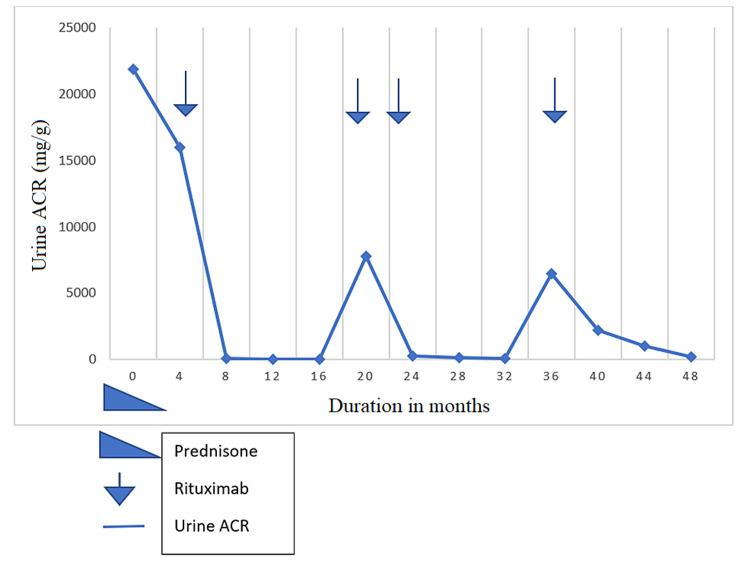
Graphic representation of urine ACR after rituximab for patient 1. ACR: albumin-creatinine ratio

A 78-year-old-female who was diagnosed with MCD initially presented with urine albumin-creatinine ratio (UACR) of 8600 mg/g. She presented with an initial albumin-creatinine ratio of 8600 mg/g. Renal pathology showed more than 50% podocyte effacement consistent with MCD. Initial treatment with prednisone 1 mg/kg with a slow taper over four months resulted in relapse with discontinuation of corticosteroids. In nine months, she had two relapses when the prednisone dose was less than 10 mg. The patient developed steroid-induced hyperglycemia so she was switched to rituximab during her third relapse as her UACR increased to 21.9 g/g. She got one dose of rituximab 375 mg/m^2^ and her UACR improved to 92 mg/g in four months. She remained in remission for 11 months. During her first relapse after rituximab, she received two doses of rituximab 375 mg/m^2^ and she went into remission for 14 months. For her second relapse, she received a dose of rituximab 375 mg/m^2^ and she is now in remission for 19 months (Figure 1).

Case 2

Again, a female patient presented with acute kidney injury with nephrotic range proteinuria (11,900 mg/g) initially requiring hemodialysis. Renal biopsy showed MCD with arterionephrosclerosis. She was on prednisone at 1 mg/kg with a slow taper over 14 weeks but started to relapse and was re-introduced on high-dose prednisone. She also developed steroid-induced hyperglycemia that required insulin. Given the adverse event with steroid dependence, she was switched to rituximab 375 mg/m^2^ (got two doses four weeks apart) and so far, remained in remission for 12 months now.

Case 3

A male patient was diagnosed with frequently relapsing but steroid-responsive MCD at the age of 20 years. He had developed multiple complications from steroid dependence including diabetes mellitus, chronic kidney disease from recurrent acute kidney injury from volume depletion, hypertension, seizures, and depression. Since then, he had been treated with other immunosuppressive agents including cyclosporine, tacrolimus, and Cellcept. Seventeen years after diagnosis of MCD, rituximab 375 mg/m^2^ (two infusions) put him in remission for nine months, which has been the longest period he has remained in remission (without steroids). During his subsequent relapse, he was again treated with two doses of rituximab and is now in remission for two years.

Case 4

Again, we have a male patient who was diagnosed with MCD at the age of 22 years with renal biopsy showing acute tubular injury, mild mesangial expansion, mesangial IgA(3+), C3 (2+), IgM (2+), IgG (1 +), and severe foot process effacement on electron microscopy. He was initially treated with prednisone 1 mg/kg but developed frequent relapses with steroid tapering. He then received a dose of rituximab of 375 mg/m^2^ after which his UACR improved from 4600 mg/g to 120 mg/g, and he has been in remission for 14 months. The baseline characteristics of the patients are shown in Table 1. 

**Table 1:** Demographics and baseline characteristics of patients. ACR: albumin-creatinine ratio; LM: light microscopy; EM: electron microscopy; ATI: acute tubular injury.

Demographics	Patient 1	Patient 2	Patient 3	Patient 4
Gender	Female	Female	Male	Male
Age at diagnosis (years)	78	69	5	22
Age at the time of rituximab infusion (years)	80	69	22	23
Duration of follow-up (years)	4	1	9	3
Serum creatinine (mg/dL) at presentation	1.2	3.6	1	1.5
Serum albumin (mg/dL) at presentation	2.3	2.6	2.6	1.8
Urine ACR (mg/g)	21,900	11,900	4200	4600
Previous Rx	Prednisone 1 mg/kg	Prednisone 1 mg/kg	Cyclosporine, tacrolimus, Ccellcept + prednisone	Prednisone
Biopsy	Normal LM, severe foot process effacement in EM	Diffuse foot process effacement superimposed on arterionephrosclerosis.	Normal LM, severe foot process effacement in EM	ATI, mild mesangial expansion, mesangial IgA (3+), C3 (2+), IgM (2+), IgG (1+), severe foot process effacement on EM
Initial rituximab regimen	1 dose of 375 mg/m^2^	2 doses of 375 mg/m^2^ 4 weeks apart	2 doses of 375 mg/m^2^	1 dose of 375 mg/m^2^
Number of relapses after initial rituximab	2	0	1	0
Second rituximab regimen	2 doses of 375 mg/m^2^	-	2 doses of 375 mg/m^2^	-
Third rituximab regimen	1 dose of 375 mg/m^2^	-	-	-
Duration of remission after the last dose of rituximab	19 months	12 months	24 months	14 months
Urine ACR (mg/g) after the last dose of rituximab	200	350	80	120

## Discussion

Dysregulation of the immune system is found to be an important factor in the pathogenesis of minimal change disease (MCD). T-cells are the main effector in this process leading to podocyte foot process effacement [9]. B-cells, in addition to their antibody-secreting properties, provide antigen and co-stimulatory signals and produce cytokines to modulate T-cell differentiation. Rituximab, in recent times, has become popular with rapid induction and prolonged remission in adult patients with MCD.

When MCD is a primary T-cell-mediated immune system dysregulation, it is intriguing that rituximab works effectively providing longer remission. Rituximab binds to CD20 on the surface of precursor and mature B-cells. It activates apoptosis via complement-dependent cytotoxicity and antibody-dependent cytotoxicity, leading to the rapid depletion of B-cells. Since B-cells activate T-helper cells through their antigen presentation, depletion of B-cells alters T-cell function [10].

The effectiveness of rituximab and its ability to maintain longer remission may lie in its potential mechanisms activating protein kinases and phospholipase Cγ, mediating inhibition of B-cell growth or leading to apoptosis. Furthermore, rituximab enhances CTLA4 produced by regulatory T-cells, which in turn inhibits CD80 activation and thus reduces proteinuria [11].

All four patients met the clinical criteria for nephrotic syndrome and all had minimal change disease proven by biopsy. In our cohort, patients 2 and 4 achieved complete remission for >12 months after two doses of rituximab 375 mg/m^2^ four weeks apart and a single dose of rituximab 375 mg/m^2^, respectively. Only patient 1 had 2 relapses after the initial rituximab but the maximum amount of proteinuria decreased with each relapse and the patient remained in remission for more than 10 months each time which was not seen with prednisone tapering.

All four patients tolerated the infusion of rituximab fairly well without any adverse effects such as infusion reactions, skin eczema, or eosinophilia [12]. Also, comparing the costs before and after rituximab treatment, the overall cost may be lower due to the longer remission period.

## Conclusions

The median rate of relapse is significantly reduced after rituximab therapy, and additional immunosuppressive exposure is minimized. The major limitations of the study are the small study population and relatively short duration of follow-up. However, rituximab can be used as a safe and effective front-line therapy for adult patients with frequently relapsing MCD, especially patients at risk of developing adverse events of corticosteroids. An ideal treatment regimen for rituximab maintenance therapy needs to be established. Furthermore, the long-term effectiveness and safety of repeated doses of rituximab need to be further investigated.

## References

[1] Waldman M, Crew RJ, Valeri A (2007). Adult minimal-change disease: clinical characteristics, treatment, and outcomes. Clin J Am Soc Nephrol.

[2] Rovin BH, Adler SG, Barratt J (2021). Executive summary of the KDIGO 2021 guideline for the management of glomerular diseases. Kidney Int.

[3] Nolasco F, Cameron JS, Heywood EF, Hicks J, Ogg C, Williams DG (1986). Adult-onset minimal change nephrotic syndrome: a long-term follow-up. Kidney Int.

[4] Liu D, Ahmet A, Ward L (2013). A practical guide to the monitoring and management of the complications of systemic corticosteroid therapy. Allergy Asthma Clin Immunol.

[5] Bargman JM (1999). Management of minimal lesion glomerulonephritis: evidence-based recommendations. Kidney Int Suppl.

[6] Nieto MF, Jayne DR (2016). Con: the use of calcineurin inhibitors in the treatment of lupus nephritis. Nephrol Dial Transplant.

[7] Selewski DT, Shah GV, Mody RJ, Rajdev PA, Mukherji SK (2010). Rituximab (Rituxan). AJNR Am J Neuroradiol.

[8] Fenoglio R, Sciascia S, Beltrame G (2018). Rituximab as a front-line therapy for adult-onset minimal change disease with nephrotic syndrome. Oncotarget.

[9] de Fátima Pereira W, Brito-Melo GE, Guimarães FT, Carvalho TG, Mateo EC, Simões e Silva AC (2014). The role of the immune system in idiopathic nephrotic syndrome: a review of clinical and experimental studies. Inflamm Res.

[10] Taguchi S, Ohtake T, Mochida Y, Ishioka K, Moriya H, Hidaka S, Kobayashi S (2020). Efficacy of repeat-dose rituximab maintenance therapy for minimal change disease in adults. Clin Exp Nephrol.

[11] Zoja C, Benigni A, Remuzzi G (2004). Cellular responses to protein overload: key event in renal disease progression. Curr Opin Nephrol Hypertens.

[12] Kasi PM, Tawbi HA, Oddis CV, Kulkarni HS (2012). Clinical review: serious adverse events associated with the use of rituximab - a critical care perspective. Crit Care.

